# Third-Eye Rivalry

**DOI:** 10.1177/2041669520927722

**Published:** 2020-07-30

**Authors:** Regan M. Gallagher, Naotsugu Tsuchiya

**Affiliations:** School of Psychological Science, Turner Institute for Brain and Mental Health, Monash University, Melbourne, Australia; School of Psychological Science, Turner Institute for Brain and Mental Health, Monash University, Melbourne, Australia; Center for Information and Neural Networks, National Institute of Information and Communications Technology, Osaka, Japan; Department of Dynamic Brain Imaging (DBI), Advanced Telecommunications Research Computational Neuroscience Laboratories, Kyoto, Japan

**Keywords:** third eye, cyclopean eye, binocular rivalry, conscious perception

## Abstract

We showcase an optical phenomenon that we call *Third-Eye Rivalry*. The effect is most easily induced by viewing one’s own reflection in a mirror. Using the pupil of the opposing eye as a fixation target, people can easily cross their eyes in free fusion to experience vivid rivalry. The resulting percept is of a prominent central “third” eye and two peripheral faces rivaling for perceptual dominance. We illustrate the process of achieving third-eye rivalry and discuss historical connotations of the third eye in scientific and mystical contexts.

Three-dimensional vision is the result of binocular fusion, which occurs when each eye receives sufficiently similar information. With effort, it is possible to change the vergence angle of the eyes and “free fuse” any two objects in the visual field in an attempt to see them as a singular object in depth. When stable fixation is achieved, and if the brain receives conflicting information from each eye, the observer can experience a vivid sense of visual alternation (i.e., binocular rivalry). The different interpretations can compete for perceptual dominance so that no percept persists indefinitely. Here, we report a condition in which sustained free fusion results in vivid binocular rivalry from the reflection of one’s own face. The effect is most easily achieved using a mirror to fixate each eye on its opposite.

In the phenomenon reported here, we found that it is rather easy to use one’s own pupils as fixation targets. If you look in the mirror and cross (or uncross) your eyes, the usually singular percept of your own face staring back at you will be doubled, resulting in two adjacent faces with four eyes distributed horizontally. If you then free fuse two of the reflected eyes, three will remain—two eyes will be perceived peripherally with a third eye seen between them. The pupil of the “middle” eye will offer a strong enough vergence cue to help maintain stable fixation. The effect and procedure are depicted in Figure 1.

**Figure 1.**
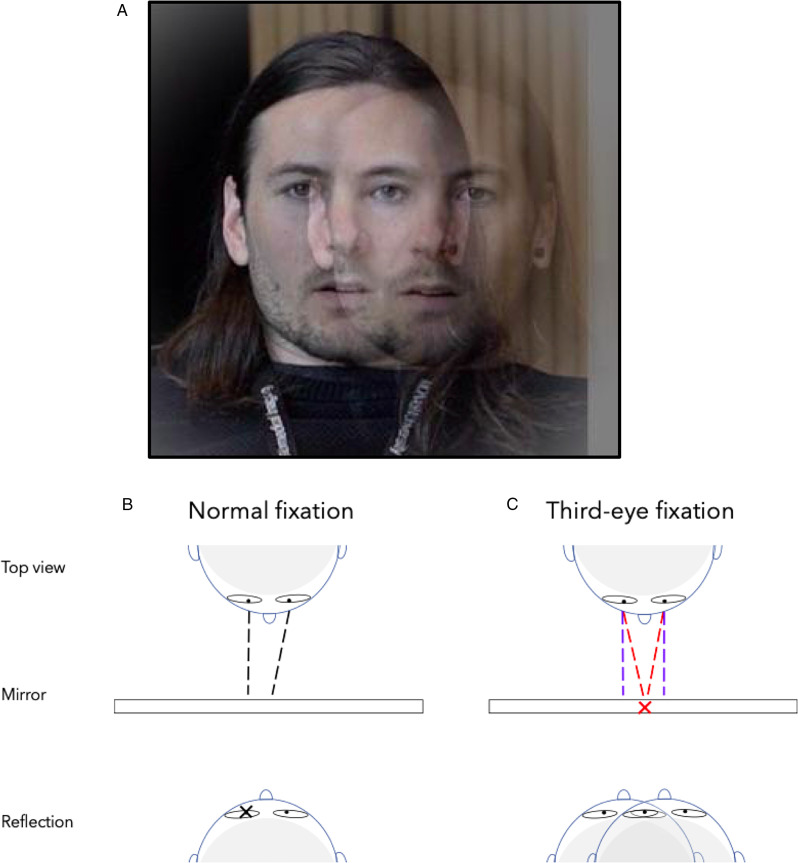
A: A “third eye” results from free-fusing reflections of the left and right eyes in a mirror. B: Normal fixation in a mirror involves binocularly viewing one pupil at a time, whereas third-eye rivalry (C) involves simultaneous fixation of both pupils, either through binocular convergence (as shown in red) or divergence (in blue).

As has long been known in the binocular rivalry literature (see Alais & Blake, 2005; Wheatstone, 1838 for a review; also Brascamp et al., 2015), discrepant monocular cues will be intermittently suppressed or dominant in awareness. In third-eye rivalry, each eye concurrently fixates the opposing eye (or by uncrossing the eyes, each eye concurrently fixates itself), and the result is the clear and stable percept of a “third eye” in the midline of the stimulus. The reflection of the subject in the mirror then becomes the object of perceptual rivalry. Phenomenologically, the observer tends to experience the following four percepts (Figure 2): two transparent face contours with three eyes (Panel A), the intermittent alternation of face dominance and suppression (Panels B and C), with periods of percepts in which only a singular eye is visible (Panel D). The last percept is reminiscent of the one-eyed cyclops from Greek mythology. From Figure 2, you can get a sense of the effect of third-eye fixation, assuming you have the ability to free fuse discordant signals after crossing your own eyes (for best results, find a mirror, or colleague, and stand as close as feasible, approximately 20–50 cm). The ability to use one’s own face as a rivaling stimulus makes the current phenomenon a potentially useful stimulus in studying the dynamics of binocular rivalry, the neuroscience of consciousness, and the perception of one’s own appearance.

**Figure 2.**
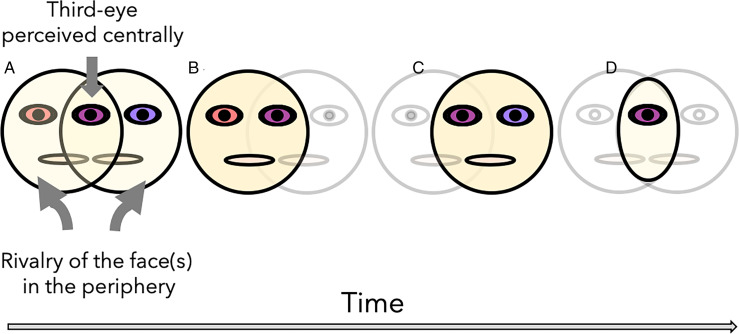
Maintaining Free Fusion of Both Pupils Concurrently in the Mirror Results in Third-Eye Rivalry. The multistable phenomenology includes (A) two face contours with three eyes; (B and C) one dominant and one suppressed face; and (D) a single central eye, as though of a cyclops.

For such a simple effect requiring only a mirror, it would be surprising if the third-eye rivalry effect had not been reported before. A search for the effect (see acknowledgments) shows no direct depictions of the phenomenon (see Sharp, 1928, for the related Frankfurter illusion), but a theoretical third eye appears in vision science under different names, such as the “cyclopean” eye (shown in Figure 3(A)), the visual egocenter, the binoculus, the projection center, and the center for visual direction (Ono & Barbeito, 1982). The concept arises from the fact that the apparent direction of an object is distorted by differences in the visual information available to each eye (Hering, 1868/1977; Ono, 1981). Binocular fusion of two images into one produces a theoretical axis of visual direction centered on a point midway between the two physical eyes. Estimating the cyclopean eye’s location usually involves fixating distant objects through an aperture or window, with subjects judging the direction or location of objects when viewed with one or both eyes (Howard & Rogers, 2012). Replacing the window with a mirror maintains the geometry of visual direction and creates the impression of two noses framing the central of three eyes, situated between two rivaling faces (shown in Figure 3(B)). Curiously, most depictions neglect the presence of the nose (which is always visible to both eyes). A sketch of the first-person perspective (Figure 3(C)) similarly shows a central vantage point framed by two noses, which suggests our phenomenon is a novel depiction of the cyclopean eye.

**Figure 3.**
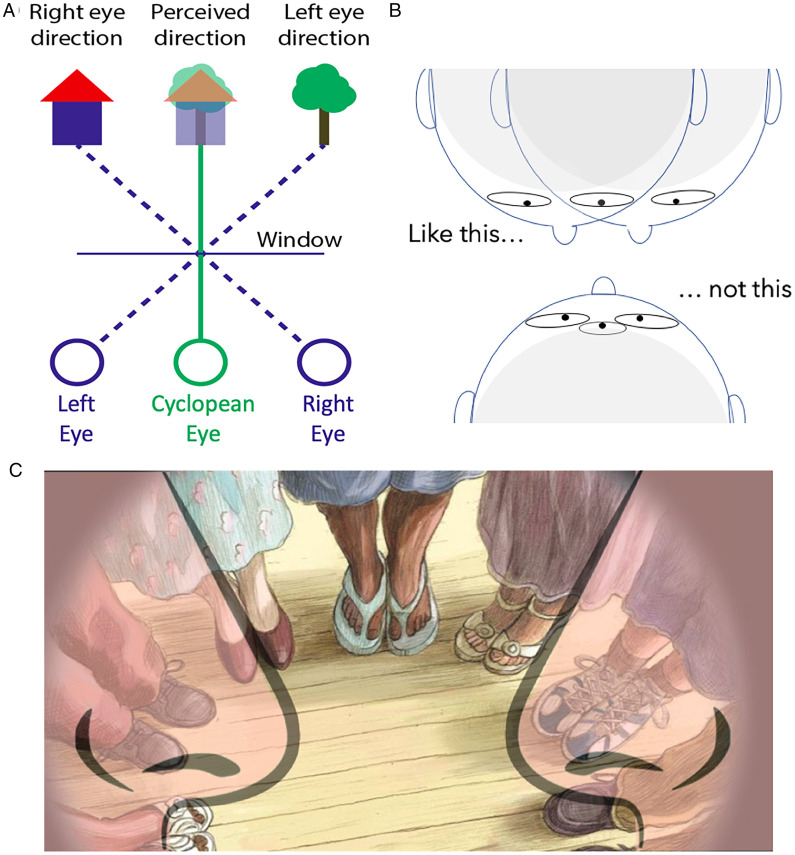
Seeing the Cyclopean Eye. A: Hering’s depiction of the cyclopean eye. B: Our phenomenon suggests that the third eye is situated between two noses (above) rather than the more intuitive interpretation (below). C: The first person viewpoint is framed by two noses (image adapted from Harding’s “On having no head: Zen and the rediscovery of the obvious,” 1998).

The third eye also commonly appears in contexts of anatomy, physiology, philosophy, spirituality, and psychedelia. In Eastern mystic traditions, for example, opening the third eye (called the Ajna chakra; Grimes, 1996; Johari, 2000) is part of the spiritual enlightenment process. The third eye symbolizes the ability to see unobstructed by the illusions of material reality, particularly as these deceptions pertain to a sense of self or ego (Mookerjee, 1984). Spontaneous third-eye opening is associated with intense and vivid intuitions or “visions,” which are described by Kundalini practitioners as profoundly unitive conscious experiences (often called “ego death,” or sometimes a psychotic break in contexts of Western psychology; see Avalon & Woodroffe, 1974; Lukoff, 1985; Sannella, 1987). To intentionally open the third eye, which is to see through “the illusion of a self,” individuals are taught to “turn the mind back on itself,” typically through intense yogic, tantric, meditative, or shamanic practice (Mookerjee, 1984, pp.123-126, p.128; Walsh, 1994). In a similar fashion, we have described a situation where the individual turns each eye on itself, leading the usually solidified, unified visual image of the self to appear illusory; the “self” disappears when looking at/from one’s own third eye.

In other contexts, biologists have identified retinal projections from a vestigial third parietal eye in some reptiles and amphibians (Su et al., 2006); modern neuroscientists and medical doctors have identified the pineal gland (the evolutionary homologue of the parietal eye) as being light sensitive in humans (Sapède & Cau, 2013), and which is hypothesized to have a pivotal function in both dreaming (Callaway, 1988) and some psychedelic experiences (Strassman, 2000); Rene Descartes pondered whether the human pineal gland provided the entry point for the soul into the body (Lokhorst, 2005) and the pineal gland is now referred to as “the third eye” by some contemporary authors (e.g., Mano & Fukada, 2007); and modern philosophers refer to the third eye as the *mind’s* eye (or the mind’s “I”; Dennett & Hofstadter, 1981). The third eye certainly carries with it varied literal and symbolic meanings, some of which are depicted in Figure 4.

**Figure 4.**
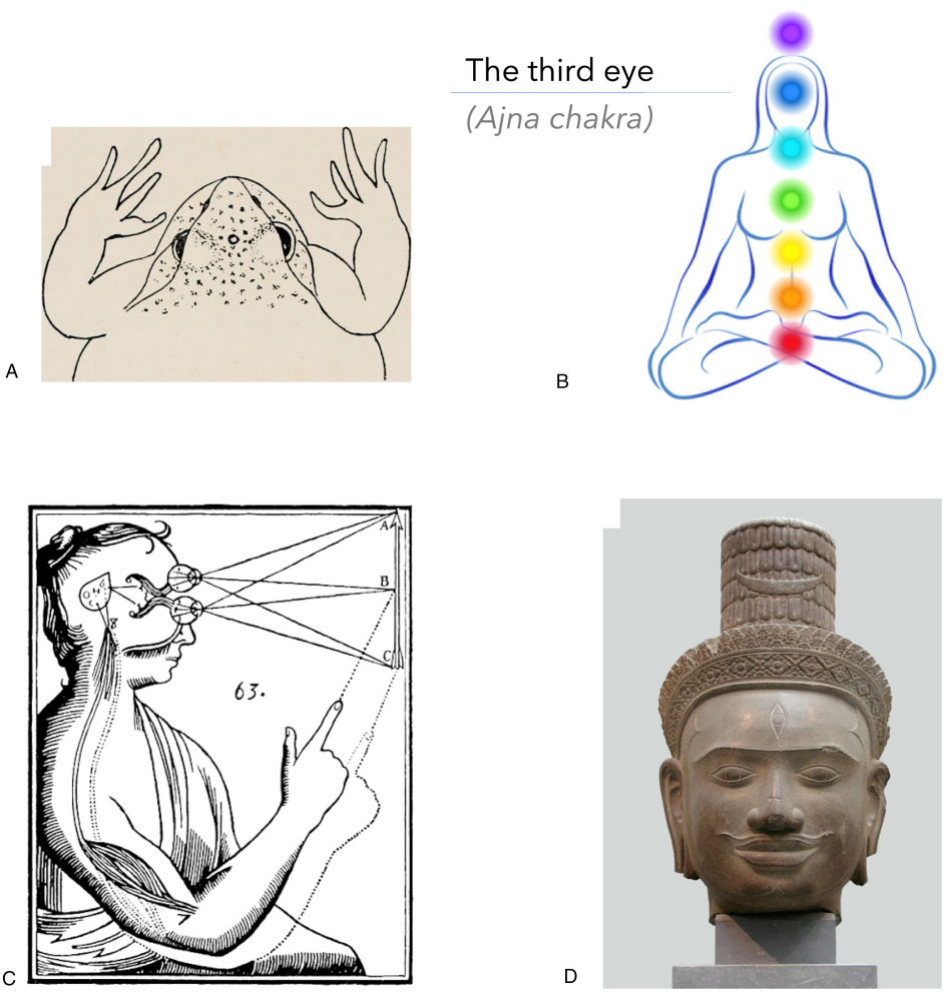
The Third Eye Depicted Through History and Across Cultures. A: The third “parietal” eye of some amphibians and reptiles. B: The Ayurvedic chakra system including the third-eye chakra. C: Rene Descartes’ depiction of the pineal gland (aka third eye) as the central relay station of consciousness (i.e., the “seat of the soul”). D: The Hindu God, Shiva, with an open third eye on the forehead.

In summary, a third eye is observed by focusing each eye on itself or its opposite in a mirror. Third-eye fixation produces the familiar aspects of binocular rivalry: wholesale and piecemeal suppression of conflicting monocular images across time. In terms of its scientific utility, binocular rivalry has often been utilized as a potent tool to study the neural correlates of conscious perception (Blake & Logothetis, 2002; Maier et al., 2012; Tong et al., 2006). As the current phenomenon invokes rivalry of one’s own face, this might have novel implications for the study of conscious experiences associated with self-perception or for understanding the role of the cyclopean eye in vision. With sustained and vivid rivalry easily inducible in untrained individuals, this could also serve as an ideal everyday rivalry demonstration. From the current observations, in which seeing the third eye dissolves the perception of self, we entertain a connection between the geometry of binocular vision and a broader set of cultural and historical depictions of the third “inner” eye.

In the scientific literature today, a persistent debate exists: when, if ever, does binocular rivalry occur in ecological contexts (Arnold, 2011; O’Shea, 2011; Shimojo & Nakayama, 1990)? To this point, this article might be informative. As with enlightenment, binocular rivalry can happen when you are brushing your teeth, bleary-eyed, at about seven in the morning.

The summary of the advice of all prophets is this; Find yourself a mirror.

—Shams Tabrizi
